# Cerebral Fat Embolism: A Rare East African Conundrum

**DOI:** 10.7759/cureus.23940

**Published:** 2022-04-07

**Authors:** Larry Mburu, Katie Du, Sylvia Mbugua, Jacqueline Mavuti, Sayed K Ali

**Affiliations:** 1 Medicine, Aga Khan University Hospital, Nairobi, KEN; 2 Internal Medicine, Aga Khan University Hospital, Nairobi, KEN; 3 Neurology, Aga Khan University Hospital, Nairobi, KEN; 4 Radiology, Aga Khan University Hospital, Nairobi, KEN

**Keywords:** long bone fractures, major trauma, east africa, fat embolism syndrome, cerebral fat embolism

## Abstract

Cerebral fat embolism (CFE) is a potentially fatal condition associated with displaced long bone fracture of the lower extremities. CFE, usually seen in young men, has an incidence ranging between 0.9% and 11% in patients with long bone fractures. CFE can present with various neurological symptoms, and a diffusion-weighted magnetic resonance imaging (MRI) (DWI) remains the definitive diagnostic study. Early treatment of the fracture is crucial in the management of CFE. To the best of our knowledge, we are the first to report a case of CFE in East Africa.

## Introduction

Fat embolism syndrome (FES) is a complication most frequently diagnosed in patients who have recently experienced long bone trauma. However, in rare cases, FES can be attributed to nontraumatic causes such as fatty liver disease, pancreatitis, osteomyelitis, sickle cell crisis, or following steroid use [1,2]. FES is characterized by the release of fat globules from fat stores, such as bone marrow, into the systemic circulation. These globules then enter the arterial system through the pulmonary vasculature and finally reach the lungs, brain, liver, kidneys, retina, and heart [2]. At these distal organ sites, fat occludes the microvasculature, leading to the initiation of the inflammatory response, subsequent platelet aggregation, and consequences such as microvasculature injury, local ischemia, and necrosis [1-4].

Diagnosing FES can be challenging given that symptoms may vary from patient to patient [4]. Not all patients may have the classical triad of respiratory distress, cerebral abnormalities, and petechiae [2,4]. FES, in a majority of cases, presents with nonspecific symptoms, such as fever, tachypnea, and tachycardia, consequently furthering diagnostic difficulty. The severity of specific and nonspecific symptoms may also vary greatly across patients. Cerebral fat embolism (CFE), a type of FES that can be challenging to diagnose, may cause only nonspecific neurological symptoms without respiratory and cutaneous manifestations [4]. CFE often follows respiratory distress; however, in cases where neurological symptoms occur in isolation, Gurd and Wilson’s diagnostic criteria (Table 1) may not be a useful tool as the criteria traditionally require two of the three features to be fulfilled for a positive diagnosis [4]. Gurd and Wilson’s criteria are most widely used in FES diagnosis, although it is neither universally accepted nor a definitive criterion for FES [2]. Similarly, other diagnostic criteria that have been developed for FES vary in their ability to diagnose FES accurately and reliably. Similar to respiratory and cutaneous symptoms of other FES types that vary in severity depending on the patient, the neurological symptoms of CFE can range from mild headaches to seizures [5]. The lack of definitive clinical features contributes to the diagnostic challenge of CFE.

Although the prognosis of most patients diagnosed with CFE is good especially if there is early intervention, the disease can be fatal and has a mortality rate of approximately 10% [6]. Therefore, early identification and correct management remain key in avoiding delays in care and minimizing mortality.

## Case presentation

A 22-year-old male patient, with no known comorbidities, presented to our institution after being hit by a motorcycle. He sustained a closed right distal tibiofibular fracture, chest wall injuries, and head trauma resulting in temporary loss of consciousness for an unquantified duration of time. At admission, his neurological examination was notable for a Glasgow Coma Scale (GCS) score of 15/15 with no neurological deficits. His initial CT scan of the head was normal. A CT scan of the chest was suggestive of a small right-sided lung contusion. An X-ray of his right leg showed a comminuted distal right tibiofibular fracture.

On the second day of his admission, he complained of headache and photophobia and was confused and agitated. His GCS progressively dropped to 10/15 over the next day. He later developed disinhibited behavior, with severe agitation and uncharacteristic foul language. His systemic examination was notable for tachypnea, tachycardia, and desaturation to 88% on room air. He also developed a generalized petechial rash, most notable on his abdomen, back, and upper extremities, which was not present before. A repeat CT was not performed.

**Figure 1:**
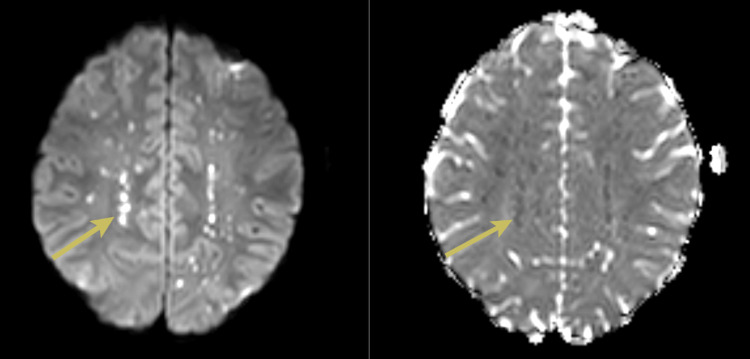
Magnetic resonance images with axial diffusion-weighted images and corresponding apparent diffusion coefficient images showing numerous punctate areas of restricted diffusion with bilateral distribution at the level of the striatum (yellow arrows)

**Figure 2:**
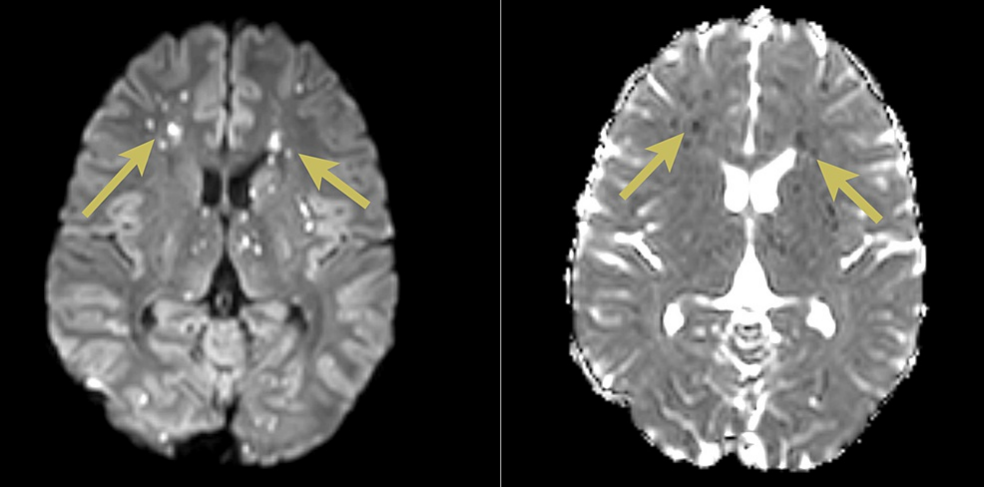
Magnetic resonance images with axial diffusion-weighted images and corresponding apparent diffusion coefficient images showing numerous punctate areas of restricted diffusion with bilateral distribution at the level of the centrum semiovale (yellow arrows)

**Figure 3:**
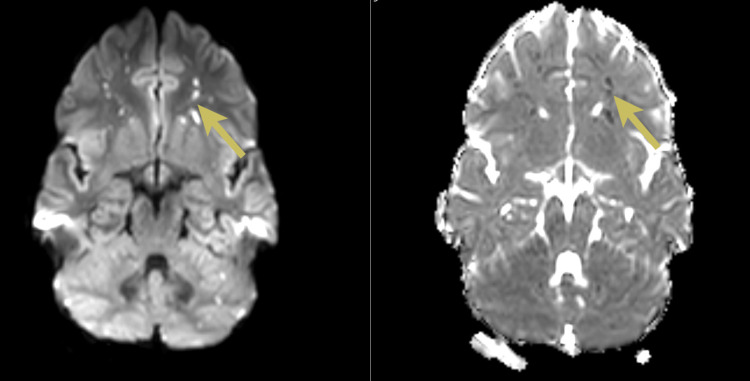
Magnetic resonance images with axial diffusion-weighted images and corresponding apparent diffusion coefficient images showing numerous punctate areas of restricted diffusion with bilateral distribution at the level of the cerebellar hemispheres (yellow arrows)

A repeat CT scan of the head revealed no new intracranial pathology. A subsequent contrast-enhanced brain magnetic resonance imaging (MRI) revealed bilateral supra- and infratentorial T2/FLAIR hyperintense foci with restricted diffusion, suggestive of embolic infarcts (Figures 1, 2, 3). MR arteriography done in the same setting revealed no evidence of carotid or aortic arch disease. Transthoracic and transesophageal echocardiography with bubble contrast were both normal, showing no shunt or thrombus in his cardiac chambers. A 24-hour Holter monitor showed no arrhythmias. An extensive autoimmune panel, including ANA, dsDNA, p-ANCA, and c-ANCA, was all negative.

The patient was managed conservatively with antipsychotic medication, including haloperidol and benzodiazepines as needed, with gradual recovery. Intramedullary nailing was done eight days post-admission, and he was discharged eight days later having made a full neurological recovery with no persisting deficits.

## Discussion

Given the fat-storing role of long bones, it is not uncommon for long bone fractures to result in the release of fat globules into the systemic circulation [8]. Usually, however, the circulating level of fat is not enough to cause FES or CFE. Various studies have reported on the incidence rates of FES and CFE after long bone fractures, and the results range from 0.9% to 11% of cases [4,9,10]. Some studies suggest that FES and CFE do not disproportionately affect either gender [4]; however, Kellogg et al. determined that the mean age of individuals diagnosed with FES or CFE was 30 years and primarily of the male gender based on a systematic review of FES and CFE patient cases reported in the literature from 1980 to 2012 [11]. The authors did not find this result to be surprising given that the distribution of severe traumatic injuries is skewed toward young men and the known association between orthopedic trauma, FES, and CFE.

Although the most common neurological presentation among FES and CFE patients is mild disorientation, approximately 20% of diagnosed individuals experience more severe neurological deficits such as hemiplegia, aphasia, agnosia, and apraxia, with one in five patients presenting with seizures [12]. These symptoms are consistent with certain autopsy results that show hemorrhaging and inflammation near petechiae sites dispersed through white matter tracts, likely caused by fat globules obstructing brain microvasculature [12-14]. Furthermore, fat emboli may localize to central areas that control catecholamine release, thereby explaining the specific association between CFE, increased catecholamine blood levels, and paroxysmal sympathetic hyperactivity (PSH) [12,15]. PSH can be clinically observed and include features such as tachycardia, hypertension, tachypnea, irregular motor tone, and hyperthermia [11].

The diagnosis of CFE relies heavily on clinical observation and magnetic resonance imaging (MRI) [4,15]. Due to the paucity of data, it is hard to tell when the incidence of CFE peaks post-trauma. Parizel et al. suggested that trauma patients admitted due to non-head-related injuries who progress from no or few neurological symptoms to becoming increasingly disorientated or presenting other signs of neurological dysfunction should be assessed for CFE [16]. The authors recommended MRI, specifically diffusion-weighted magnetic resonance imaging (DWI), as an early diagnostic tool because DWI can clearly detect the numerous microemboli in the brain characteristic of CFE with high contrast. The “starfield pattern” on brain MRI in the context of other symptoms such as changes in behavior, PSH, and petechial rash, as well as medical histories such as recent fractures or orthopedic surgery, would strongly support a CFE diagnosis. Even with the use of neuroimaging, it may still be difficult to make a definitive diagnosis as other conditions may cause microembolism and similar symptoms [4,15]. Head CT may present signs of diffuse cerebral edema, but this is usually only in the most severe cases [1,5].

Although there is no standardized clinical management guideline for CFE, early intervention can reduce the risk of disease development [1,4]. An example of an effective preventative measure is quick immobilization, stabilization, and fixation of long bone fractures in patients who have this injury [4,15]. Hyperbaric oxygen therapy has also been shown to be an effective treatment as it can improve microcirculation and the availability of oxygen to brain tissue, thereby decreasing the risk of severe neurological symptoms [4,17]. Unfortunately, until recently, hyperbaric chambers were not available in Kenya. Other measures that may be effective in preventing cerebral ischemia include treating patients with drugs that decrease cell metabolism in the brain, sedative drugs, and antiepileptic drugs, especially among patients with CFE who develop seizures. There are also more controversial measures that have varying efficacies and success. For example, corticosteroids have been used for their ability to stabilize cell membranes and decrease inflammation caused by increasing circulatory levels of free fatty acids, but Bederman et al. found that there is no significant link between corticosteroid use and improved patient outcomes [1,4,17,18]. Lipid-soluble drugs, such as saponin, which can dissolve fat emboli, have also been used as treatments [4,17]. Like corticosteroid therapy, however, the effectiveness of the approach is unclear.

## Conclusions

Our patient presented to the clinic two weeks post-discharge and reported no complaints at all. He remained oriented to time, place, and person, with a good recollection of his previous accident. His mother reported no neurological deficits and no additional inappropriate behavior. He was working diligently with physical therapy to improve his gait and balance. CFE, often presenting with various neurological symptoms, remains a rare entity seen mostly in men. The lack of definitive clinical features contributes to the diagnostic challenge of CFE. Therefore, early identification and correct management remain key in avoiding delays in care and minimizing mortality.

## Appendices

Table 1 presents Gurd and Wilson's diagnostic criteria for fat embolism syndrome.

**Table 1:** Gurd and Wilson’s criteria for FES Two major criteria or one major and four minor suggest a diagnosis of FES [7].

Major criteria
Respiratory distress
Cerebral symptoms in non-head injury patients
Petechial rash
Minor criteria
Tachycardia (>110 bpm)
Fever (>38.5°C)
Jaundice
Renal changes
Retinal changes
Drop in hemoglobin
New-onset thrombocytopenia
Elevated ESR
Fat macroglobulinemia
